# Vaginal Microbiome: Environmental, Biological, and Racial Influences on Gynecological Health Across the Lifespan

**DOI:** 10.1111/aji.70026

**Published:** 2024-12-13

**Authors:** Eleni Dubé‐Zinatelli, Luna Cappelletti, Nafissa Ismail

**Affiliations:** ^1^ NISE Laboratory School of Psychology Faculty of Social Science University of Ottawa Ottawa Ontario Canada; ^2^ LIFE Research Institute University of Ottawa Ottawa Ontario Canada; ^3^ University of Ottawa Brain and Mind Research Institute Ottawa Ontario Canada

**Keywords:** bacteria, hormones, *Lactobacillus* species, vaginal ecosystem, vaginal microbiome, vaginal microbiota

## Abstract

The human vaginal microbiome (VMB) is a complex and unique ecosystem composed of various microorganisms, including bacteria, fungi, archaea, viruses, and candidate phyla radiation. A healthy VMB is often characterized by the presence of *Lactobacillus* species, which play a crucial role in protecting and maintaining homeostasis within the vaginal environment. When this balance is disrupted, the protection of the vaginal epithelium weakens, leading to a reduction in *Lactobacillus* species and an increased risk of various gynecological and reproductive health issues. However, this generalized description can lead to misconceptions and an incomplete understanding of vaginal health, as *Lactobacillus* is not always dominant across all ages and racialized groups. Therefore, this review provides a comprehensive analysis of the environmental, biological, and racial influences on the VMB at each stage of a woman's life, highlighting their implications for gynecological health and offering a holistic understanding of the VMB for all women.

## 1 Defining a Healthy Vaginal Microbiome (VMB)

Much of the literature defines a healthy human VMB as being characterized by a predominant presence of the bacterial species *Lactobacillus* [1, 2]. Specifically, *Lactobacillus crispatus* (*L. crispatus), Lactobacillus iners* (*L. iners), Lactobacillus gasseri (L. gasseri), or Lactobacillus jensenii (L. jensenii)* [1, 2]. These species maintain vaginal homeostasis by producing metabolites such as lactate, antimicrobials, hydrogen peroxide, and lactic acid; enhancing mucus viscosity; trapping pathogens; and preventing adhesion, growth, and DNA damage to vaginal epithelial cells [1, 2, 3, 4]. However, while this generally applies to women of reproductive age, defining a “healthy vaginal microbiome” solely by the abundance of *Lactobacillus* provides a generalized and limited description. It excludes considerations for women outside of reproductive age and overlooks variations in vaginal bacterial composition across racialized groups. Additionally, not all *Lactobacillus* species are associated with a healthy VMB or the maintenance of favorable gynecological and obstetric outcomes [4, 5, 6]. For example, while *L. crispatus* is consistently linked to low genital inflammation and a reduced risk of adverse outcomes*, L. iners* is frequently associated with an unstable microbiome [5, 6, 7]. Therefore, acknowledging environmental, biological, and racial influences is essential when defining a healthy VMB to gain a more inclusive understanding.

### 1.1 Prepubescence to Menarche

While the VMB composition of prepubertal girls has received little empirical attention, most research indicates that during childhood, the VMB is highly diverse, dominated by anaerobes including *Bacteroides, Prevotella, Actinomyces*, *and Fusobacterium*, as well as some aerobic bacteria like *Staphylococcus aureus* and *Staphylococcus epidermidis* [8, 9, 10]. Similarly, a recent study that performed sequencing analysis of the vaginal flora of healthy preschool girls found that the microbiota was characterized by gram‐negative bacilli such as *Pseudomonas*, *Methylobacterium*, and *Escherichia* [11]. Moreover, the presence of these microorganisms and lack of *Lactobacillus* contribute to a neutral vaginal pH state during childhood [9, 11, 12].

As menarche approaches, increasing estradiol levels trigger the thickening of the vaginal epithelium and the production of glycogen [13, 14, 15]. Glycogen is broken down by α‐amylase into maltose, maltotriose, and α‐dextrins, which *Lactobacillus* then metabolizes into lactic acid, creating a more acidic environment [13, 15, 16]. Consequently, during puberty, the VMB becomes increasingly dominated by *Lactobacillus*, mirroring the bacterial composition of reproductive‐aged women [16, 17]. However, after menarche, the vaginal pH remains higher than what is considered healthy for reproductive‐aged women, indicating that lactobacilli levels do not reach adult counts until later in development [16]. These maturational changes collectively help inhibit the growth of bacteria such as *Gardnerella vaginalis* and other pathogens associated with the onset of sexual activity [18, 19].

### 1.2 Reproductive Age

During the reproductive age, *Lactobacillus* dominates the VMB, and that is crucial for maintaining vaginal health [20, 21, 22]. The presence of lactobacilli is significantly influenced by hormonal changes, with bacterial composition fluctuating in response to estradiol levels during different phases of the menstrual cycle [16, 23, 24]. For instance, during periods of elevated estradiol, such as the follicular, preovulatory, and midluteal phases, glycogen accumulation in the vaginal epithelium promotes lactobacilli colonization, leading to higher lactobacilli counts [23, 25]. Conversely, menstruation is associated with low estradiol levels, decreased lactobacilli concentration, and increased bacterial diversity [23, 25]. Interestingly, these alterations are more pronounced in naturally cycling women compared to those using combined oral contraceptive pills or levonorgestrel intrauterine devices, suggesting that synthetic hormones interfere with the normal variations in the VMB across the menstrual cycle [23, 24].

Another hormone that influences the VMB is cortisol, which may inhibit epithelial maturation and glycogen accumulation, leading to a reduction in *Lactobacillus* [13, 26]. The impact of psychological stress through HPA axis activation and cortisol production may explain previous findings that African American and Hispanic women have reduced lactobacilli counts and increased levels of other bacterial species, such as *Gardnerella, Sneathia, Prevotella, Megasphaera* sp.*, and Prevotella*, in comparison to White and Asian women [8, 13, 27, 28, 29]. Lower *Lactobacillus* dominance among these women compared to White women has also been linked to lower socioeconomic status, education level, and poorer access to healthcare [27, 30, 31]. While the exact reasons for racialized differences in VMB composition remain contested and deserve further exploration, these findings indicate that the impact of race on VMB is likely better explained by a variety of factors rather than by socially constructed racial categories [28, 30, 31, 32].

#### 1.2.1 Pregnancy

During a healthy pregnancy, the placenta generates estradiol and estriol, leading to increased *Lactobacillus* levels and decreased vaginal pH [33, 34, 35]. The maintenance of a stable lactobacilli population, supported by an acidic pH throughout pregnancy, protects the mother and fetus against infections [36, 37]. Furthermore, the vaginal bacterial composition of pregnant women differs from that of nonpregnant women, with higher levels of *Bacteroidales, and Clostridiales* found in pregnant samples, while nonpregnant women typically have higher *Prevotella, Streptococcus*, and *Veillonellaceae* presence [34, 36, 37]. Nevertheless, this prenatal transition to lower bacterial diversity and higher *Lactobacillus* levels has been observed to be less pronounced among Black women, potentially due to having higher rates of maternal stress [27, 38].

Following delivery, estradiol levels significantly drop, causing a decrease in lactic acid‐producing bacteria and an increase in other taxa, such as *Gardnerella vaginalis *and *Prevotella bivia*, rendering the VMB more vulnerable to pathogens [38, 39, 40]. Such changes arise as early as the onset of labor and persist for up to 1‐year postpartum [34, 40]. Physiological changes during labor and delivery have also been shown to alter vaginal composition postdelivery [34, 41]. For example, elevated levels of heat shock protein 70, as well as hyaluronan, induce changes in the properties of vaginal epithelial cells and immune cells, altering the composition of the vaginal fluid postpartum [34, 42, 43]. Similarly, among a sample of 230 women who required pelvic floor examination postpartum, participants with weakened pelvic floor function showed the highest *Lactobacillus* depletion 6–8 weeks following delivery, further supporting the impact of physiological changes on the VMB [41].

### 1.3 Menopause

The onset of menopause is marked by a decline in estrogen levels and is associated with a decrease in *Lactobacillus* concentration, lower glycogen, and an increase in vaginal pH [8, 12]. Additionally, women typically display high concentrations of anaerobic bacteria such as *Gardnerella vaginalis, Candida albicans, and Prevotella* spp. at this stage, resulting in a VMB composition comparable to prepubescence [16, 44]. Subsequently, these changes can induce thinning of the vaginal epithelium, leading to symptoms such as vaginal dryness and discomfort [45, 46, 47]. Although this process is natural with vaginal aging, estrogen treatments may be sought to increase hormone production and facilitate glycogen in the VMB, leading to enhanced vaginal mucosa [47, 48]. Conversely, the therapeutic benefit of *Lactobacillus* during menopause remains contested, as findings on the effects of continued *Lactobacillus* dominance during this life stage are mixed [46, 47, 48, 49]. Further research is needed to understand why the continued production of estrogens, but not lactobacilli, appears to promote vaginal health after menopause.

**FIGURE 1:**
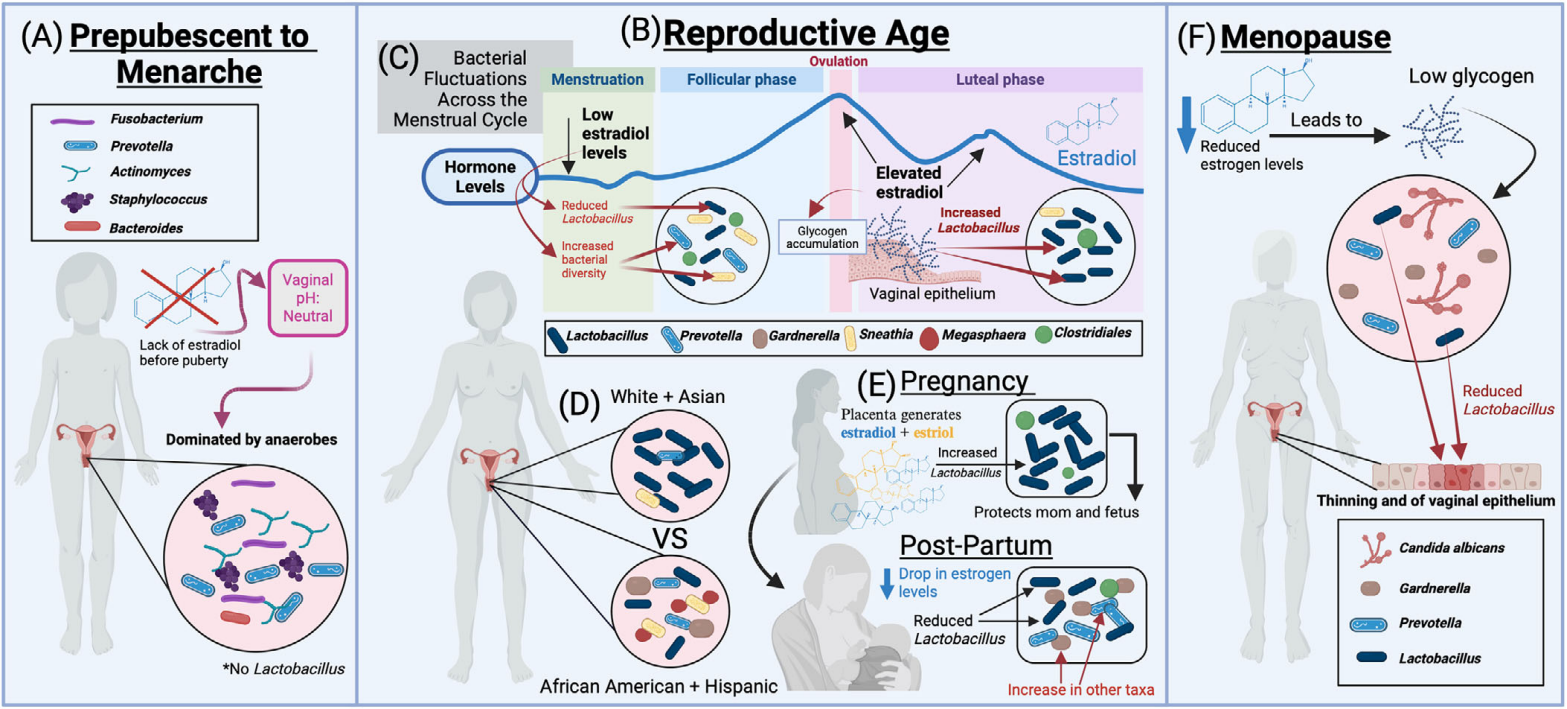
During (A) prepubescence to menarche, the absence of estradiol results in a neutral vaginal pH dominated by anaerobic bacteria, with no presence of *Lactobacillus*. As women transition into (B) reproductive age, hormonal fluctuations, particularly estradiol, drive significant changes in bacterial diversity. Throughout (C) the menstrual cycle, low estradiol levels increase bacterial diversity and reduce *Lactobacillus*, while rising estradiol levels promote *Lactobacillus* dominance. (D) Racial variations also influence the vaginal microbiota, with distinct differences in composition observed among White, Asian, African American, and Hispanic women. (E) Pregnancy introduces further changes, as elevated estradiol and estriol levels increase *Lactobacillus* to protect both the mother and fetus. However, the drop in estrogen levels postpartum leads to a reduction in *Lactobacillus* and a rise in other microbial taxa. During (F) menopause, reduced estrogen levels lead to decreased glycogen, thinning of the vaginal epithelium, and a decline in *Lactobacillus*, while species such as *Candida albicans, Prevotella*, and *Gardnerella* become more prominent. Created in BioRender.Dubé‐Zinatelli, E. (2024)

Limited data exist on the VMB composition of postmenopausal Black women. However, a recent study found that *L. crispatus and L. gasseri* dominance were significantly more prevalent in African American postmenopausal women compared to their White age‐matched counterparts, which appears to contradict findings from studies on women of reproductive age [8, 46, 50]. This highlights the need for a deeper exploration into the parameters of a healthy microbiome specifically tailored to both racial and age‐related differences (Figure 1).

## 2 Gynecological Conditions Across the Lifespan

There are numerous gynecological conditions that arise throughout a woman's life [9, 27, 48]. While many conditions predominantly affect women of reproductive age due to environmental and physical factors that increase susceptibility during this period, vaginal health is a concern for women of all ages [12, 21]. Understanding the conditions that affect women at specific stages of life is essential for comprehensive gynecological care [49, 51]. However, research on the VMB remains underdeveloped, with many discrepancies in the literature and a lack of clear definitions for specific conditions. Therefore, the following section aims to clarify these terms and provide a comprehensive understanding of the most common gynecological conditions affecting women at different life stages and their implications for health and well‐being.

### 2.1 Vaginal Dysibosis

With the rise in microbiome research, the term “dysbiosis” has become well‐established, referring to imbalances in resident microbial communities in the gut that are associated with adverse human health and disease states [52, 53]. While gut dysbiosis remains the most commonly studied type of dysbiosis, advancements in VMB research have introduced the concept of “vaginal dysbiosis” (VD). This term describes a disruption in the microbial balance of the vagina, characterized by a decrease in protective *Lactobacillus* species, which weakens the vaginal epithelium's defense and allows for pathogenic growth [3, 54].

VD is not an actual illness but rather refers to an imbalance in the VMB. However, this imbalance can lead to gynecological infections such as human papillomavirus (HPV), herpes, and human immunodeficiency virus (HIV) [3]. Conversely, these gynecological infections can also lead to a state of VD, creating a cyclical relationship where each can cause the other [3]. Additionally, much of the literature often uses VD interchangeably with bacterial vaginosis (BV), a gynecological condition characterized by a polymicrobial imbalance. Some researchers use the term VD more broadly to refer to any vaginal condition caused by a decreased prevalence of *Lactobacillus*, which includes conditions like atrophic vaginitis that are not caused by pathogens or infections [7]. Other papers define VD as encompassing all conditions that result in an altered composition of vaginal microbiota, including bacteria, viruses, and fungi. Consequently, conditions such as desquamative inflammatory vaginitis, vaginal candidiasis, and trichomoniasis are also considered types of VD [55].

Nevertheless, in almost all cases, VD typically includes only gynecological conditions that occur after puberty, as it is almost always linked to *Lactobacillus*. The issue with this definition is that the predominance of *Lactobacillus* within the VMB does not always indicate a healthy state, nor does its absence always signify dysbiosis. This is evident before puberty and in some racialized groups [9, 38]. Therefore, defining VD solely by the absence of lactobacilli is inadequate for an inclusive understanding of women's health [56, 57, 58, 59].

### 2.2 Vaginitis

The term “vaginitis,” sometimes referred to as vulvovaginitis, is commonly used in clinical settings by both patients and healthcare providers as a diagnostic term [60]. While much of the literature highlights vaginitis as a prevalent gynecological issue affecting women of all ages, it is important to understand that vaginitis is a nonspecific term encompassing a broad range of conditions that cause vulvovaginal symptoms [60]. These symptoms include abnormal vaginal discharge, odor, irritation, itching, or burning [61, 62]. Furthermore, vaginitis can be a symptom of various gynecological issues, including infectious conditions such as vulvovaginal candidiasis (VVC) and trichomoniasis, as well as noninfectious vulvar disorders like vaginal atrophy [60] (Table 1).

**TABLE 1:** Gynecological conditions across the lifespan.

Gynecological risk factors	Common conditions	Symptoms	Treatment
Prepubescence Anatomical factors (underdeveloped labia minora and majora) Physiological factors (low vaginal pH) Behavioral factors (hygiene practices like nose‐picking, thumb‐sucking) *All leading to the intrusion of pathogenic microorganisms*	Nonspecific vulvovaginitis	Inflammation of the vulvar and vaginal regions.	Educating and improving vulvar hygiene (e.g., teaching proper wiping techniques, avoiding tight or irritating clothing) Sitz baths to soothe symptoms and clean the area without using soap or washcloth
	Lichen sclerosis (inflammatory skin condition)	Pain, pruritus, and a burning sensation along the perineal region.	High‐dose topical steroids with gradual transition to lower‐potency steroids
	Pinworms (infection)	Usually asymptomatic, but can include itching of the anal or vaginal area, especially at night, vaginal discharge, and insomnia.	Antiparasitic such as pyrantel pamoate
Reproductive age Variations in innate and adaptive immune systems Increased sexual activity and partners Shifts in estrogen Antibiotic use Psychosocial stress Racial and ethnic differences	Bacterial vaginosis	Unusual vaginal discharge, odor, itching, and burning sensations.	Antibiotics such as metronidazole and clindamycin
	Trichomoniasis (sexually transmitted infection)	Vaginal discharge (often yellow or green), painful intercourse, urinary tract infection symptoms, vaginal itching, and pelvic pain.	Antibiotics such as metronidazole and clindamycin
	Vulvovaginal candidiasis (fungal infection)	Vulvar and vaginal erythema, excoriations, thick white adherent discharge, vaginal itching, burning, pain and redness and swelling.	Antifungals such as fluconazole
Menopause Drop in estradiol Rise in vaginal pH Loss of lactobacilli Overgrowth of pathogenic bacteria	Vulvovaginal atrophy	Vaginal dryness, decreased lubrication, pruritus, soreness, stinging pain in the vaginal and vulvar areas, vaginal spotting, thin yellow or grey watery discharge, increased urinary frequency, urgency, and incontinence.	Hormone replacement therapy (HRT)

#### 2.2.1 Prepubescence

“Vulvovaginitis” is typically used to refer to the most common gynecological condition affecting prepubescent girls. Many studies simply refer to it as vulvovaginitis, but it is also known as prepubertal vulvovaginitis or nonspecific vulvovaginitis [63, 64]. Nonspecific vulvovaginitis accounts for 61.8% of the gynecological problems observed in children and adolescents [63, 65]. In pediatric patients, around 70%–80% of cases of vulvovaginitis have nonspecific causes and are defined quite broadly by inflammation of the vulvar and vaginal regions [9, 11, 63].

This condition is generally associated with anatomical, physiological, and behavioral factors linked to prepubescence leading to the intrusion of pathogenic microorganisms [9, 66]. Prepubertal females exhibit an underdeveloped labia minora and a labia majora with minimal adipose tissue and a lack of pubic hair, increasing the susceptibility of the vagina to bacterial intrusion from the anus and a higher risk of irritation [67]. Moreover, the absence of estrogen production during this phase results in an alkaline vaginal pH and decreased protective lactobacilli concentration, creating an environment conducive to the overgrowth of fecal or oropharyngeal bacteria [68]. Lastly, young girls often exhibit less‐than‐optimal hygiene practices such as nose‐picking, thumb‐sucking, or scratching, coupled with inadequate handwashing, which facilitates the transfer of oropharyngeal and skin bacteria to the vaginal area, increasing their vulnerability to vulvovaginitis [66, 67].

Prepubescent vulvovaginitis is complex due to the controversial nature of what constitutes normal vaginal flora at this age [51, 69, 70]. This uncertainty makes it difficult to identify the presence of pathogens, determine the causes, and select the appropriate treatment [70]. However, in cases of prepubescent vulvovaginitis where foreign bodies or sexually transmitted infections (STIs) are found, the possibility of sexual abuse must be considered, and appropriate measures are subsequently taken [21, 71, 72].

The causes of vulvovaginitis in prepubertal patients differ from those in pubertal patients. A study comparing vaginal samples from prepubertal and pubertal girls found that prepubertal girls predominantly had gram‐positive *cocci and Enterobacteriaceae*, while VVC and BV were more commonly detected in adolescents [72]. These differences are due to lactobacilli starting to populate the vagina with the onset of estrogen production during puberty, which explains why estrogen‐dependent Candida species are quite unusual before puberty [72]. Additionally, the higher rate of sexual activity during adolescence contributes to the prevalence of BV and Candida infections compared to childhood [73]. In some cases, Candida may occur in infants and toddlers still in diapers or in patients with predisposing risk factors such as recent antibiotic use, underlying immunosuppression, or a diagnosis of diabetes [70, 73].

Given that the vaginal microbiology present during childhood is different from that of adulthood, using the typical treatments for adult vaginitis has been shown to be ineffective and exacerbate symptoms in children [9, 11, 74]. Currently, when the causes of the condition are known, most treatments revolve around educating and improving vulvar hygiene, such as teaching children to wipe front to back and avoiding tight or irritating clothing. Sitz baths are also recommended to soothe symptoms and help keep the area clean without using soaps or washcloths [67, 74]. In cases where pathogens are present, such as pinworms, antiparasitics are used for treatment [67]. For inflammatory skin conditions like lichen sclerosis, high‐dose topical steroids are initially used, with a gradual transition to lower‐potency steroids for maintenance [70, 73]. Taken together, vulvovaginitis, particularly nonspecific vulvovaginitis, is most common among prepubescent girls, with most of the causes remaining unknown. This lack of understanding complicates treatment and highlights the need for more research on the VMB of girls at this age. Improving knowledge of the VMB and the mechanism behind these conditions is crucial for better prevention and treatment, as gynecological health during childhood may have lasting effects into adulthood.

#### 2.2.2 Reproductive Age

Infectious vaginitis is most common in women of reproductive age [75]. The primary causes are BV, VVC, and trichomoniasis [76]. VVC, a fungal infection caused mostly by the polymorphic opportunistic fungus *Candida albicans*, affects 70%–75% of women over their lifetimes in the United States [77, 78, 79]. Typical clinical features include vulvar and vaginal erythema, excoriations, thick white adherent discharge, vaginal itching, burning, pain, and redness and swelling [77, 80, 81]. *Candida* are asymptomatic species that are part of the VMB of reproductive age women and can later develop pathogenicity [79, 80, 81]. Trichomoniasis, one of the most prevalent nonviral STIs in the United States, is caused by the protozoan *Trichomonas vaginalis* [82]. Although it affects both women and men, 89% of trichomoniasis patients are women [82]. In women, clinical features include vaginal discharge (often yellow or green), painful intercourse, urinary tract infection symptoms, vaginal itching, and pelvic pain [83, 84]. BV is the most common condition of the lower genital tract, affecting approximately 30% of females of reproductive age. It is characterized by a decrease in *Lactobacillus* species, leading to a shift from *Lactobacillus* dominance to an increased abundance of anaerobic species such as *Gardnerella*, *Leptotrichia*, *Prevotella*, and facultative bacteria [3, 29]. Symptoms of BV include unusual vaginal discharge, odor, itching, and burning sensations [29, 85, 86].

While each of these conditions has unique characteristics, they will be grouped together to provide a general overview of how they affect women during the reproductive period. Generally, all three conditions arise from disruptions in the balance of the vaginal microbiota, weakening the protection of the vaginal epithelium and allowing for a shift in microbial communities [3]. Consequently, protective *Lactobacillus* species are replaced by pathogenic microorganisms, leading to various gynecological and reproductive health issues [2, 3, 40, 57, 87]. Multiple factors can lead to these conditions, most of which are linked to endogenous and exogenous changes during the reproductive period, such as shifts in estrogen levels, increased sexual activity and partners, and pregnancy [20]. Other causes include differences in gene polymorphisms, variations in the innate and adaptive immune systems, the composition and quantity of vaginal secretions, and epithelial cell surface ligands, allergies, antibiotic use, serum glucose levels, and psychosocial stress [4, 29, 79–81]. For VVC, the antifungal fluconazole is the first‐line treatment [78]. BV and trichomoniasis are often treated with oral antibiotics such as metronidazole and clindamycin [81, 88–90].

Importantly, sexual practices contribute to the development of all three conditions, with increased sexual partners increasing susceptibility [91, 92]. Additionally, male sexual partners of women with these conditions can affect recurrence rates by being asymptomatic and unknowingly spreading the infection [93, 94, 95]. As a result, treating asymptomatic male partners can enhance women's cure rates for trichomoniasis [81]. Conversely, in the case of BV, treating male sexual partners with oral metronidazole has shown no effect on recurrence in the female [96]. For acute VVC, treatment is not recommended for asymptomatic sexual partners or women who are asymptomatic [93].

During reproductive age, these conditions are most prevalent and have been shown to pose significant obstetric issues affecting both mother and fetus, such as increased maternal and fetal/infant morbidity, amniotic fluid infection, preterm labor, and premature birth [29, 87, 97–99]. A common problem with the standard antibiotic treatment is the risk of antibiotic resistance and health risks they pose for the developing fetus during pregnancy [100, 101, 102]. Interestingly, probiotic treatments for vaginal infections have recently emerged as promising alternatives to avoid antibiotic resistance and harm to the fetus. These treatments work by restoring protective *Lactobacillus* to the VMB [103, 104, 105].

Racial and ethnic differences significantly impact the risk of acquiring vaginal infections [91, 106, 107]. For example, African American women report symptoms more frequently and with higher incidence than White women [106]. African American women also have a higher risk of contracting BV compared to White or Asian American women, with prevalence rates among African American women being twice as high as that of White women [8, 86, 108]. Moreover, *Trichomonas vaginalis* disproportionately affects Black women [91, 94]. Reasons for these variations may include racial differences in bacterial composition, which affect susceptibility to gynecological infections [8, 86]. However, more research is needed to understand these implications since factors such as access to healthcare, lower socioeconomic status, and higher levels of stress in racialized women have also been seen to contribute to increased gynecological infections in Black women [28, 30, 31, 32, 107].

#### 2.2.3 Menopause

There are also gynecological conditions leading to vaginitis that do not involve infections. One such condition is atrophic vaginitis, also known as vulvovaginal atrophy (VVA), which affects menopausal women. VVA, often defined as a collection of symptoms associated with vaginal dryness, is a common condition that affects 50%–60% of postmenopausal females [46, 109]. VVA occurrence has been linked to estradiol levels dropping during menopause, which induces physiological and structural changes within the vulvovaginal mucosa [22, 110].

Prior to menopause, estrogens induce the proliferation of the vaginal epithelium wall layers, resulting in smooth muscle fibers, collagen maintaining moist and thick vaginal epithelium, and the presence of rugae [46, 111]. However, declining levels of estrogens during menopause can lead to the thinning of the vaginal epithelium, less exfoliation of cells into the vagina, and a rise of vaginal pH between 5 and 7.5, ultimately resulting in a loss of lactobacilli and an overgrowth of pathogenic bacteria such as staphylococci, group B streptococci, and coliforms [110, 112].

After menopause, a reduction in the elasticity of the vagina, an increase in connective tissue, a decrease in vaginal blood flow, and a decrease in vaginal lubrication are all common symptoms but can also be associated with early stages of VVA [46, 110, 112]. As VVA persists, vaginal dryness occurs, and thinning of the epithelial lining leads to pruritus, soreness, and stinging pain in the vaginal and vulvar areas [111]. Vaginal spotting, due to small tears in the vaginal epithelium, may also occur, as well as some thin yellow or grey watery discharge from the rise of pH [109]. Urinary symptoms associated with VVA include increased frequency, urgency, and incontinence [111]. Due to minimal changes in the vaginal mucosa, clinical manifestations of VVA are reported in only 4% of women during the first few years following menopause [109]. As estrogen deficiency grows, dystrophic and atrophic changes develop in the vaginal mucosa, vulva, and structures of the urogenital tract, where atrophic changes are observed in almost 50% of women 7–10 years after the end of menstruation, eventually affecting 73%–75% of women [22, 109]. Given the association between estrogen depletion, reduction in *Lactobacillus* in the VMB, and subsequent VVA, it is unclear why some research has found that postmenopausal women continue to have stable lactobacilli presence in the absence of natural hormones, although the absence of the menstrual cycle may play a supporting role in VMB stability [46, 50]. Given the potential influence of birth control and other synthetic hormones on the VMB [36, 37], future research must control for hormone replacement therapy (HRT) use among postmenopausal women in order to make this relationship clearer.

Standard treatment for VVA often consists of HRT, as the administration of estrogens locally or orally can help improve glycogen production and *Lactobacillus* abundance in the VMB [47, 48, 113]. However, the need for nonhormonal treatments is dire, given that antiestrogens are used as a preventative treatment in breast cancer survivors, and these treatments often result in or exacerbate VVA symptoms [114, 115, 116]. A comprehensive overview of VVA therapeutics and the impact of reproductive cancers is outside of the scope of this paper, although several recent nonhormonal treatments have shown promise [115, 116, 117].

## 3 Conclusion

The VMB, with its primary domination of *Lactobacillus* species during reproductive age, plays a pivotal role in preserving vaginal health and protecting against infections. While a *Lactobacillus* dominant vaginal environment is commonly associated with a healthy microbiome, it is essential to recognize that *Lactobacillus* dominance as a universal indicator of vaginal health does not account for differences observed across age, racialized groups, and hormonal fluctuation. Very little research has focused on the VMB of women outside of reproductive age, making it difficult to draw conclusions on distinct bacterial changes. Given the observed racial differences in VMB composition among reproductive‐aged women, it would be interesting to investigate whether these differences are present before puberty.

Evidence suggests considerable variation in the VMB throughout the lifespan, implying that gynecological consequences will manifest differently across each developmental phase. Thus, it is crucial to understand how environmental, biological, and racial influences affect the VMB at each stage and their implications for health and well‐being.

## Ethics Statement

The authors confirm that the ethical policies of the journal, as noted on the journal's author guidelines page, have been adhered to. No ethical approval was required as this is a review article with no original research data.

## Conflicts of Interest

The authors declare no conflicts of interest.

## Data Availability

The data that support the findings of this study are available from the corresponding author upon reasonable request. The data are not publicly available due to privacy or ethical restrictions.

## References

[1] A. Bhattacharya , S. Das , M. J. Bhattacharjee , A. K. Mukherjee , and M. R. Khan , “Comparative Pangenomic Analysis of Predominant Human Vaginal Lactobacilli Strains Towards Population‐Specific Adaptation: Understanding the Role in Sustaining a Balanced and Healthy Vaginal Microenvironment,” BMC Genomics [Electronic Resource] 24, no. 1 (2023): 565, 10.1186/s12864-023-09665-y. 37740204 PMC10517566

[2] K. Diop , J.‐C. Dufour , A. Levasseur , and F. Fenollar , “Exhaustive Repertoire of Human Vaginal Microbiota,” Human Microbiome Journal 11 (2019): 100051, 10.1016/j.humic.2018.11.002.

[3] Y. Han , Z. Liu , and T. Chen , “Role of Vaginal Microbiota Dysbiosis in Gynecological Diseases and the Potential Interventions,” Frontiers in Microbiology 12 (2021): 643422, 10.3389/fmicb.2021.643422. 34220737 PMC8249587

[4] J. Ravel , P. Gajer , Z. Abdo , et al., “Vaginal Microbiome of Reproductive‐Age Women,” PNAS 108, no. S1 (2011): 4680–4687, 10.1073/pnas.1002611107. 20534435 PMC3063603

[5] M. N. Anahtar , D. B. Gootenberg , C. M. Mitchell , and D. S. Kwon , “Cervicovaginal Microbiota and Reproductive Health: The Virtue of Simplicity,” Cell Host & Microbe 23, no. 2 (2018): 159–168, 10.1016/j.chom.2018.01.013. 29447695

[6] B. J. Callahan , D. B. DiGiulio , D. S. A. Goltsman , et al., “Replication and Refinement of a Vaginal Microbial Signature of Preterm Birth in Two Racially Distinct Cohorts of US Women,” PNAS 114, no. 37 (2017): 9966–9971, 10.1073/pnas.1705899114. 28847941 PMC5604014

[7] J. H. H. M. van De Wijgert and V. Jespers , “The Global Health Impact of Vaginal Dysbiosis,” Research in Microbiology 168, no. 9–10 (2017): 859–864, 10.1016/j.resmic.2017.02.003. 28257809

[8] L. Lehtoranta , R. Ala‐Jaakkola , A. Laitila , and J. Maukonen , “Healthy Vaginal Microbiota and Influence of Probiotics Across the Female Life Span,” Frontiers in Microbiology 13 (2022): 819958, 10.3389/fmicb.2022.819958. 35464937 PMC9024219

[9] W. Xiaoming , L. Jing , P. Yuchen , L. Huili , Z. Miao , and S. Jing , “Characteristics of the Vaginal Microbiomes in Prepubertal Girls With and Without Vulvovaginitis,” European Journal of Clinical Microbiology & Infectious Diseases 40, no. 6 (2021): 1253–1261, 10.1007/s10096-021-04152-2. 33452946 PMC8139898

[10] J. A. Younes , E. Lievens , R. Hummelen , R. Van Der Westen , G. Reid , and M. I. Petrova , “Women and Their Microbes: The Unexpected Friendship,” Trends in Microbiology 26, no. 1 (2018): 16–32, 10.1016/j.tim.2017.07.008. 28844447

[11] Y. Zhang , T. Liu , J. Lin , F. Yu , and Z. Hu , “STROBE‐Sequencing Analysis of the Vaginal Microecology of 4‐ to 6‐Year‐Old Girls in Southwest China,” Medicine 100, no. 13 (2021): e25362, 10.1097/MD.0000000000025362. 33787640 PMC8021340

[12] R. S. Auriemma , R. Scairati , G. Del Vecchio , et al., “The Vaginal Microbiome: A Long Urogenital Colonization Throughout Woman Life,” Frontiers in Cellular and Infection Microbiology 11 (2021): 686167, 10.3389/fcimb.2021.686167. 34295836 PMC8290858

[13] E. Amabebe and D. O. Anumba , “Psychosocial Stress, Cortisol Levels, and Maintenance of Vaginal Health,” Frontiers in Endocrinology 9 (2018): 568, 10.3389/fendo.2018.00568. 30319548 PMC6165882

[14] J. D. Blaustein , N. Ismail , and M. K. Holder , “Review: Puberty as a Time of Remodeling the Adult Response to Ovarian Hormones,” Journal of Steroid Biochemistry and Molecular Biology 160 (2016): 2–8, 10.1016/j.jsbmb.2015.05.007. 26004504 PMC4654988

[15] N. Deka , S. Hassan , G. Seghal Kiran , and J. Selvin , “Insights Into the Role of Vaginal Microbiome in Women's Health,” Journal of Basic Microbiology 61, no. 12 (2021): 1071–1084, 10.1002/jobm.202100421. 34763361

[16] R. J. Hickey , X. Zhou , M. L. Settles , et al., “Vaginal Microbiota of Adolescent Girls Prior to the Onset of Menarche Resemble Those of Reproductive‐Age Women,” mBio 6, no. 2 (2015): e00097–15, 10.1128/mBio.00097-15. PMC4453513 25805726

[17] T. Curley and C. S. Forster , “Recurrent UTIs in Girls: What Is the Role of the Microbiome?” Urology 151 (2021): 94–97, 10.1016/j.urology.2020.04.091. 32389817

[18] M. J. Bruins , C. O. Dos Santos , R. A. M. J. Damoiseaux , and G. J. H. M. Ruijs , “Bacterial Agents in Vulvovaginitis and Vaginal Discharge: A 10‐Year Retrospective Study in the Netherlands,” European Journal of Clinical Microbiology & Infectious Diseases 40, no. 10 (2021): 2123–2128, 10.1007/s10096-021-04265-8. 33942163

[19] V. Jespers , L. Hardy , J. Buyze , J. Loos , A. Buvé , and T. Crucitti , “Association of Sexual Debut in Adolescents With Microbiota and Inflammatory Markers,” Obstetrics and Gynecology 128, no. 1 (2016): 22–31, 10.1097/AOG.0000000000001468. 27275789

[20] X. Chen , Y. Lu , T. Chen , and R. Li , “The Female Vaginal Microbiome in Health and Bacterial Vaginosis,” Frontiers in Cellular and Infection Microbiology 11 (2021): 631972, 10.3389/fcimb.2021.631972. 33898328 PMC8058480

[21] H. Kaur , M. Merchant , M. M. Haque , and S. S. Mande , “Crosstalk Between Female Gonadal Hormones and Vaginal Microbiota Across Various Phases of Women's Gynecological Lifecycle,” Frontiers in Microbiology 11 (2020): 551, 10.3389/fmicb.2020.00551. 32296412 PMC7136476

[22] A. L. Muhleisen and M. M. Herbst‐Kralovetz , “Menopause and the Vaginal Microbiome,” Maturitas 91 (2016): 42–50, 10.1016/j.maturitas.2016.05.015. 27451320

[23] M. C. Krog , L. W. Hugerth , E. Fransson , et al., “The Healthy Female Microbiome Across Body Sites: Effect of Hormonal Contraceptives and the Menstrual Cycle,” Human Reproduction 37, no. 7 (2022): 1525–1543, 10.1093/humrep/deac094. 35553675 PMC9247429

[24] S. Lebeer , S. Ahannach , T. Gehrmann , et al., “A Citizen‐Science‐Enabled Catalogue of the Vaginal Microbiome and Associated Factors,” Nature Microbiology 8, no. 11 (2023): 2183–2195, 10.1038/s41564-023-01500-0. PMC10627828 37884815

[25] J. P. Brooks , D. J. Edwards , D. L. Blithe , et al., “Effects of Combined Oral Contraceptives, Depot Medroxyprogesterone Acetate and the Levonorgestrel‐Releasing Intrauterine System on the Vaginal Microbiome,” Contraception 95, no. 4 (2017): 405–413, 10.1016/j.contraception.2016.11.006. 27913230 PMC5376524

[26] A. M. Holdcroft , D. J. Ireland , and M. S. Payne , “The Vaginal Microbiome in Health and Disease—What Role Do Common Intimate Hygiene Practices Play?” Microorganisms 11, no. 2 (2023): 298, 10.3390/microorganisms11020298. 36838262 PMC9959050

[27] K. McKee , C. M. Bassis , J. Golob , et al., “Host Factors Are Associated With Vaginal Microbiome Structure in Pregnancy in the ECHO Cohort Consortium,” Scientific Reports 14, no. 1 (2024): 11798, 10.1038/s41598-024-62537-7. 38782975 PMC11116393

[28] H. Borgdorff , C. Van Der Veer , R. Van Houdt , et al., “The Association Between Ethnicity and Vaginal Microbiota Composition in Amsterdam, the Netherlands,” PLoS ONE 12, no. 7 (2017): e0181135, 10.1371/journal.pone.0181135. 28700747 PMC5507447

[29] A. S. Mondal , R. Sharma , and N. Trivedi , “Bacterial Vaginosis: A State of Microbial Dysbiosis,” Medical Microbiology 16, no. 2 (2023): 100082, 10.1016/j.medmic.2023.100082.

[30] M. Dixon , A. L. Dunlop , E. J. Corwin , and M. R. Kramer , “Joint Effects of Individual Socioeconomic Status and Residential Neighborhood Context on Vaginal Microbiome Composition,” Frontiers in Public Health 11 (2023): 1029741, 10.3389/fpubh.2023.1029741. 36761121 PMC9902942

[31] W. A. Grobman , J. L. Bailit , M. M. Rice , et al., “Racial and Ethnic Disparities in Maternal Morbidity and Obstetric Care,” Obstetrics and Gynecology 125, no. 6 (2015): 1460–1467, 10.1097/AOG.0000000000000735. 26000518 PMC4443856

[32] H. Onywera , A.‐L. Williamson , Z. Z. A. Mbulawa , D. Coetzee , and T. L. Meiring , “Factors Associated With the Composition and Diversity of the Cervical Microbiota of Reproductive‐Age Black South African Women: A Retrospective Cross‐Sectional Study,” Peer Journal 7 (2019): e7488, 10.7717/peerj.7488. PMC6698374 31435492

[33] P. Gupta , M. P. Singh , and K. Goyal , “Diversity of Vaginal Microbiome in Pregnancy: Deciphering the Obscurity,” Frontiers in Public Health 8 (2020): 326, 10.3389/fpubh.2020.00326. 32793540 PMC7393601

[34] K. L. Nunn , S. S. Witkin , G. M. Schneider , et al., “Changes in the Vaginal Microbiome During the Pregnancy to Postpartum Transition,” Reproductive Sciences 28, no. 7 (2021): 1996–2005, 10.1007/s43032-020-00438-6. 33432532 PMC8189965

[35] A. L. Prince , D. M. Chu , M. D. Seferovic , K. M. Antony , J. Ma , and K. M. Aagaard , “The Perinatal Microbiome and Pregnancy: Moving Beyond the Vaginal Microbiome. Cold Spring Harb,” Perspectives in Medicine 5, no. 6 (2015): a023051–a023051, 10.1101/cshperspect.a023051. 25775922 PMC4448707

[36] A. Baud , K.‐H. Hillion , C. Plainvert , et al., “Microbial Diversity in the Vaginal Microbiota and Its Link to Pregnancy Outcomes,” Scientific Reports 13, no. 1 (2023): 9061, 10.1038/s41598-023-36126-z. 37271782 PMC10239749

[37] S. Shabayek , A. M. Abdellah , M. Salah , M. Ramadan , and N. Fahmy , “Alterations of the Vaginal Microbiome in Healthy Pregnant Women Positive for Group B Streptococcus Colonization During the Third Trimester,” BMC Microbiology 22 (2022): 313, 10.1186/s12866-022-02730-8. 36544085 PMC9769055

[38] K. T. Li , F. Li , H. Jaspan , et al., “Changes in the Vaginal Microbiome During Pregnancy and the Postpartum Period in South African Women: A Longitudinal Study,” Reproductive Sciences 31, no. 1 (2024): 275–287, 10.1007/s43032-023-01351-4. 37721699 PMC10784382

[39] L. Abou Chacra and F. Fenollar , “Exploring the Global Vaginal Microbiome and Its Impact on Human Health,” Microbial Pathogenesis 160 (2021): 105172, 10.1016/j.micpath.2021.105172. 34500016

[40] C. Chopra , I. Bhushan , M. Mehta , et al., “Vaginal Microbiome: Considerations for Reproductive Health,” Future Microbiology 17 (2022): 1501–1513, 10.2217/fmb-2022-0112. 36314380

[41] Y. Zhang , H. Yang , L. Lin , W. Yang , G. Xiong , and G. Gao , “The Relationship Between Pelvic Floor Functions and Vaginal Microbiota in 6–8 Weeks Postpartum Women,” Frontiers in Microbiology 13 (2022): 975406, 10.3389/fmicb.2022.975406. 36406409 PMC9669797

[42] M. Mahendroo , “Cervical Hyaluronan Biology in Pregnancy, Parturition and Preterm Birth,” Matrix Biology 78–79 (2019): 24–31, 10.1016/j.matbio.2018.03.002. PMC6120812 29510230

[43] N. Saghafi , L. Pourali , V. Ghavami Ghanbarabadi , F. Mirzamarjani , and M. Mirteimouri , “Serum Heat Shock Protein 70 in Preeclampsia and Normal Pregnancy: A Systematic Review and Meta‐Analysis,” International Journal of Reproductive BioMedicine 16, no. 1 (2018): 1–8. 29707695 PMC5899764

[44] M. G. Park , S. Cho , and M. M. Oh , “Menopausal Changes in the Microbiome—A Review Focused on the Genitourinary Microbiome,” Diagnostics 13, no. 6 (2023): 1193, 10.3390/diagnostics13061193. 36980501 PMC10047399

[45] C. Foschi , S. Alvisi , M. Baldassarre , et al., “Vaginal Metabolites in Postmenopausal Women With or Without Vulvo‐Vaginal Atrophy at Baseline and After Ospemifeme and Systemic Hormone Treatment,” Maturitas 159 (2022): 7–14, 10.1016/j.maturitas.2021.12.007. 35337615

[46] R. Hummelen , J. M. Macklaim , J. E. Bisanz , et al., “Vaginal Microbiome and Epithelial Gene Array in Post‐Menopausal Women With Moderate to Severe Dryness,” PLoS ONE 6, no. 11 (2011): e26602, 10.1371/journal.pone.0026602. 22073175 PMC3206802

[47] K. Tomczyk , K. Chmaj‐Wierzchowska , K. Wszołek , and M. Wilczak , “New Possibilities for Hormonal Vaginal Treatment in Menopausal Women,” Journal of Clinical Medicine 12, no. 14 (2023): 4740, 10.3390/jcm12144740. 37510854 PMC10380877

[48] J. Shen , N. Song , C. J. Williams , et al., “Effects of Low Dose Estrogen Therapy on the Vaginal Microbiomes of Women With Atrophic Vaginitis,” Scientific Reports 6 (2016): 24380, 10.1038/srep24380. 27103314 PMC4840317

[49] J. K. Szymański , A. Słabuszewska‐Jóźwiak , and G. Jakiel , “Vaginal Aging—What We Know and What We Do Not Know,” International Journal of Environmental Research and Public Health 18, no. 9 (2021): 4935, 10.3390/ijerph18094935. 34066357 PMC8125346

[50] P. L. Hudson , W. Ling , M. C. Wu , et al., “Comparison of the Vaginal Microbiota in Postmenopausal Black and White Women,” Journal of Infectious Diseases 224, no. 11 (2021): 1945–1949, 10.1093/infdis/jiaa780. 33367735 PMC8825215

[51] H. Verstraelen , P. Vieira‐Baptista , F. De Seta , G. Ventolini , R. Lonnee‐Hoffmann , and A. Lev‐Sagie , “The Vaginal Microbiome: I. Research Development, Lexicon, Defining ‘Normal’ and the Dynamics throughout Women's Lives,” Journal of Lower Genital Tract Disease 26, no. 1 (2022): 73–78, 10.1097/LGT.0000000000000643. 34928256 PMC8719517

[52] K. B. Hooks and M. A. O'Malley , “Dysbiosis and Its Discontents,” mBio 8, no. 5 (2017): e01492–17, 10.1128/mBio.01492-17. PMC5635691 29018121

[53] S. E. Winter and A. J. Bäumler , “Gut Dysbiosis: Ecological Causes and Causative Effects on Human Disease,” PNAS 120, no. 50 (2023): e2316579120, 10.1073/pnas.2316579120. 38048456 PMC10722970

[54] X. Chen , J. Wang , J. Chen , G. Wang , R. Zhang , and J. Qiu , “Vaginal Homeostasis Features of Vulvovaginal Candidiasis Through Vaginal Metabolic Profiling,” Medical Mycology 61, no. 8 (2023): myad085, 10.1093/mmy/myad085. 37573133

[55] T. Wrønding , K. Vomstein , E. F. Bosma , et al., “Antibiotic‐free Vaginal Microbiota Transplant With Donor Engraftment, Dysbiosis Resolution and Live Birth After Recurrent Pregnancy Loss: A Proof of Concept Case Study,” EClinicalMedicine 61 (2023): 102070, 10.1016/j.eclinm.2023.102070. 37528843 PMC10388571

[56] A. Ansari , D. Son , Y. M. Hur , et al., “Lactobacillus Probiotics Improve Vaginal Dysbiosis in Asymptomatic Women,” Nutrients 15, no. 8 (2023): 1862, 10.3390/nu15081862. 37111086 PMC10143682

[57] A. Machado , C. Foschi , and A. Marangoni , “Editorial: Vaginal Dysbiosis and Biofilms,” Frontiers in Cellular and Infection Microbiology 12 (2022): 976057, 10.3389/fcimb.2022.976057. 36017371 PMC9396345

[58] S. Swidsinski , W. Maria Moll , and A. Swidsinski , “Bacterial Vaginosis—Vaginal Polymicrobial Biofilms and Dysbiosis,” Deutsches Arzteblatt International 120, no. 20 (2023): 347–354, 10.3238/arztebl.m2023.0090. 37097068 PMC10412922

[59] J. H. H. M. van de Wijgert , “The Vaginal Microbiome and Sexually Transmitted Infections Are Interlinked: Consequences for Treatment and Prevention,” PloS Medicine 14, no. 12 (2017): e1002478, 10.1371/journal.pmed.1002478. 29281632 PMC5744905

[60] P. Nyirjesy , “Management of Persistent Vaginitis,” Obstetrics and Gynecology 124, no. 6 (2014): 1135–1146, 10.1097/AOG.0000000000000551. 25415165

[61] M. E. Egan and M. S. Lipsky , “Diagnosis of Vaginitis,” American Family Physician 62 (2000): 1095–1104. 10997533

[62] J. P. Hildebrand and A. T. Kansagor , Vaginitis (St. Petersburg, FL: StatPearls Publishing, 2024). 29262024

[63] E. P. A. Brander and S. K. McQuillan , “Prepubertal Vulvovaginitis,” CMAJ: Canadian Medical Association Journal 190, no. 26 (2018): E800, 10.1503/cmaj.180004. 29970369 PMC6028267

[64] C. Templeman and J. S. Sanfilippo . “Chapter 8 ‐ Pediatric and Adolescent Gynecology,” in General Gynecology, eds. A. I. Sokol and E. R. Sokol (Maryland Heights, Missouri: Mosby, 2007), 187–204, 10.1016/B978-032303247-6.10008-5.

[65] E. E. Koumantakis , E. A. Hassan , E. K. Deligeoroglou , and G. K. Creatsas , “Vulvovaginitis During Childhood and Adolescence,” Journal of Pediatric and Adolescent Gynecology 10, no. 1 (1997): 39–43, 10.1016/S1083-3188(97)70043-3. 9061634

[66] İ. Beyitler and S. Kavukcu , “Clinical Presentation, Diagnosis and Treatment of Vulvovaginitis in Girls: A Current Approach and Review of the Literature,” World Journal of Pediatrics 13, no. 2 (2017): 101–105, 10.1007/s12519-016-0078-y. 28083751

[67] A. Zuckerman and M. Romano , “Clinical Recommendation: Vulvovaginitis,” Journal of Pediatric and Adolescent Gynecology 29, no. 6 (2016): 673–679, 10.1016/j.jpag.2016.08.002. 27969009

[68] F. Cemek , D. Odabaş , Ü. Şenel , and A. T. Kocaman , “Personal Hygiene and Vulvovaginitis in Prepubertal Children,” Journal of Pediatric and Adolescent Gynecology 29, no. 3 (2016): 223–227, 10.1016/j.jpag.2015.07.002. 26187769

[69] G. Ranđelović , V. Mladenović , L. Ristić , et al., “Microbiological Aspects of Vulvovaginitis in Prepubertal Girls,” European Journal of Pediatrics 171, no. 8 (2012): 1203–1208, 10.1007/s00431-012-1705-9. 22383074

[70] M. E. Romano , “Prepubertal Vulvovaginitis,” Clinical Obstetrics and Gynecology 63, no. 3 (2020): 479–485, 10.1097/GRF.0000000000000536. 32282354

[71] J. F. Anders , “Pediatric Gynecologic Disorders,” in Emergency Medicine, ed. J. G. Adams (Amsterdam, the Netherlands: Elsevier, 2013), 177–184.e1, 10.1016/B978-1-4377-3548-2.00021-5.

[72] S. Baka , S. Demeridou , G. Kaparos , et al., “Microbiological Findings in Prepubertal and Pubertal Girls With Vulvovaginitis,” European Journal of Pediatrics 181, no. 12 (2022): 4149–4155, 10.1007/s00431-022-04631-4. 36163515 PMC9649474

[73] M. Loveless and O. Myint , “Vulvovaginitis‐ Presentation of More Common Problems in Pediatric and Adolescent Gynecology,” Best Practice & Research. Clinical Obstetrics & Gynaecology 48 (2018): 14–27, 10.1016/j.bpobgyn.2017.08.014. 28927766

[74] S. E. Vilano and C. L. Robbins , “Common Prepubertal Vulvar Conditions,” Current Opinion in Obstetrics & Gynecology 28 no. 5 (2016): 359–365, 10.1097/GCO.0000000000000309. 27517340

[75] H. Mujuzi , A. Siya , and R. Wambi , “Infectious Vaginitis Among Women Seeking Reproductive Health Services at a Sexual and Reproductive Health Facility in Kampala, Uganda,” BMC Womens Health 23 no. 1 (2023): 677, 10.1186/s12905-023-02835-w. 38114988 PMC10729507

[76] A. Bitew and Y. Abebaw , “Vulvovaginal Candidiasis: Species Distribution of Candida and Their Antifungal Susceptibility Pattern,” BMC Womens Health 18, no. 1 (2018): 94, 10.1186/s12905-018-0607-z. 29902998 PMC6003188

[77] R. Jeanmonod , V. Chippa , and D. Jeanmonod , Vaginal Candidiasis (St. Petersburg, FL: StatPearls Publishing, 2024). 29083806

[78] M. Satora , A. Grunwald , B. Zaremba , et al., “Treatment of Vulvovaginal Candidiasis—An Overview of Guidelines and the Latest Treatment Methods,” Journal of Clinical Medicine 12, no. 16 (2023): 5376, 10.3390/jcm12165376. 37629418 PMC10455317

[79] H. M. E. Willems , S. S. Ahmed , J. Liu , Z. Xu , and B. M. Peters , “Vulvovaginal Candidiasis: A Current Understanding and Burning Questions,” Journal of Fungi 6, no. 1 (2020): 27, 10.3390/jof6010027. 32106438 PMC7151053

[80] H. F. Jenkinson and L. J. Douglas , “Interactions Between Candida Species and Bacteria in Mixed Infections,” in Polymicrobial Diseases, eds. K. A. Brogden and J. M. Guthmiller (Washington, DC: ASM Press, 2002). 21735561

[81] J. Van Schalkwyk , M. H. Yudin , M. H. Yudin , et al., “Vulvovaginitis: Screening for and Management of Trichomoniasis, Vulvovaginal Candidiasis, and Bacterial Vaginosis,” Journal of Obstetrics and Gynaecology Canada 37, no. 3 (2015): 266–274, 10.1016/S1701-2163(15)30316-9. 26001874

[82] H.‐C. Lin , K.‐Y. Huang , C.‐H. Chung , et al., “Infection With Trichomonas Vaginalis Increases the Risk of Psychiatric Disorders in Women: A Nationwide Population‐Based Cohort Study,” Parasites & Vectors 12, no. 1 (2019): 88, 10.1186/s13071-019-3350-x. 30867042 PMC6417068

[83] M. F. Rein , “Trichomoniasis,” Hunter's Tropical Medicine and Emerging Infectious Diseases (Amsterdam, the Netherlands: Elsevier, 2020), 731–733, 10.1016/B978-0-323-55512-8.00100-9.

[84] J. A. Schumann and S. Plasner , Trichomoniasis (St. Petersburg, FL: StatPearls Publishing, 2024). 30521247

[85] N. Kairys and M. Garg , Bacterial Vaginosis (St. Petersburg, FL: StatPearls Publishing, 2024). 29083654

[86] V. S. Saraf , S. A. Sheikh , A. Ahmad , P. M. Gillevet , H. Bokhari , and S. Javed , “Vaginal Microbiome: Normalcy Vs Dysbiosis,” Archives of Microbiology 203, no. 7 (2021): 3793–3802, 10.1007/s00203-021-02414-3. 34120200

[87] A. Lev‐Sagie , F. De Seta , H. Verstraelen , G. Ventolini , R. Lonnee‐Hoffmann , and P. Vieira‐Baptista , “The Vaginal Microbiome: II. Vaginal Dysbiotic Conditions,” Journal of Lower Genital Tract Disease 26, no. 1 (2022): 79–84, 10.1097/LGT.0000000000000644. 34928257 PMC8719518

[88] L. Abou Chacra , F. Fenollar , and K. Diop , “Bacterial Vaginosis: What Do We Currently Know?” Frontiers in Cellular and Infection Microbiology 11 (2022): 672429. 35118003 10.3389/fcimb.2021.672429 PMC8805710

[89] C. S. Bradshaw and J. D. Sobel , “Current Treatment of Bacterial Vaginosis—Limitations and Need for Innovation,” Journal of Infectious Diseases 214, no. S1 (2016): S14–S20, 10.1093/infdis/jiw159. 27449869 PMC4957510

[90] P. J. Kissinger , C. A. Gaydos , A. C. Seña , et al., “Diagnosis and Management of *Trichomonas Vaginalis*: Summary of Evidence Reviewed for the 2021 Centers for Disease Control and Prevention Sexually Transmitted Infections Treatment Guidelines,” Clinical Infectious Diseases 74, no. S2 (2022): S152–S161, 10.1093/cid/ciac030. 35416973 PMC9006969

[91] E. U. Patel , C. A. Gaydos , Z. R. Packman , T. C. Quinn , and A. A. R. Tobian , “Prevalence and Correlates of Trichomonas Vaginalis Infection Among Men and Women in the United States,” Clinical Infectious Diseases 67, no. 2 (2018): 211–217, 10.1093/cid/ciy079. 29554238 PMC6031067

[92] S. M. Rogers , C. F. Turner , M. Hobbs , et al., “Epidemiology of Undiagnosed Trichomoniasis in a Probability Sample of Urban Young Adults,” PLoS ONE 9 (2014): e90548, 10.1371/journal.pone.0090548. 24626058 PMC3953116

[93] A. Farr , I. Effendy , B. Frey Tirri , et al., “Guideline: Vulvovaginal Candidosis (AWMF 015/072, Level S2k),” Mycoses 64, no. 6 (2021): 583–602, 10.1111/myc.13248. 33529414 PMC8248160

[94] E. Meites , C. A. Gaydos , M. M. Hobbs , et al., “A Review of Evidence‐Based Care of Symptomatic Trichomoniasis and Asymptomatic *Trichomonas Vaginalis* Infections,” Clinical Infectious Diseases 61, no. S8 (2015): S837–S848, 10.1093/cid/civ738. 26602621 PMC4657597

[95] O. T. Van Gerwen , A. F. Camino , J. Sharma , P. J. Kissinger , and C. A. Muzny , “Epidemiology, Natural History, Diagnosis, and Treatment of *Trichomonas Vaginalis* in Men,” Clinical Infectious Diseases 73, no. 6 (2021): 1119–1124, 10.1093/cid/ciab514. 34079999 PMC8522801

[96] J. R. Schwebke , S. Y. Lensing , J. Lee , et al., “Treatment of Male Sexual Partners of Women With Bacterial Vaginosis: A Randomized, Double‐Blind, Placebo‐Controlled Trial,” Clinical Infectious Diseases of Public Infectious Diseases Society of America 73, no. 3 (2020): e672–e679, 10.1093/cid/ciaa1903. PMC8326574 33383580

[97] M. F. Cotch , J. G. Pastorek , R. P. Nugent , et al., “Trichomonas Vaginalis Associated With Low Birth Weight and Preterm Delivery,” Sexually Transmitted Diseases 24, no. 6 (1997): 353–360, 10.1097/00007435-199707000-00008. 9243743

[98] B. K. Ng , J. N. Chuah , F. C. Cheah , et al., “Maternal and Fetal Outcomes of Pregnant Women With Bacterial Vaginosis,” Frontiers in Surgery 10 (2023): 1084867, 10.3389/fsurg.2023.1084867. 36860946 PMC9968788

[99] B. J. Silver , R. J. Guy , J. M. Kaldor , M. S. Jamil , and A. R. Rumbold , “Trichomonas Vaginalis as a Cause of Perinatal Morbidity: A Systematic Review and Meta‐Analysis,” Sexually Transmitted Diseases 41, no. 6 (2014): 369–376, 10.1097/OLQ.0000000000000134. 24825333

[100] C. R. Cohen , M. R. Wierzbicki , A. L. French , et al., “Randomized Trial of Lactin‐V to Prevent Recurrence of Bacterial Vaginosis,” New England Journal of Medicine 382, no. 20 (2020): 1906–1915, 10.1056/NEJMoa1915254. 32402161 PMC7362958

[101] F. Minooei , N. M. Gilbert , L. Zhang , et al., “Rapid‐Dissolving Electrospun Nanofibers for Intra‐Vaginal Antibiotic or Probiotic Delivery,” European Journal of Pharmaceutics and Biopharmaceutics 190 (2023): 81–93, 10.1016/j.ejpb.2023.07.009. 37479065 PMC10530173

[102] P. Nyirjesy , C. Brookhart , G. Lazenby , J. Schwebke , and J. D. Sobel , “Vulvovaginal Candidiasis: A Review of the Evidence for the 2021 Centers for Disease Control and Prevention of Sexually Transmitted Infections Treatment Guidelines,” Clinical Infectious Diseases 74, no. S2 (2022): S162–S168, 10.1093/cid/ciab1057. 35416967

[103] R. Chen , R. Li , W. Qing , et al., “Probiotics Are a Good Choice for the Treatment of Bacterial Vaginosis: A Meta‐Analysis of Randomized Controlled Trial,” Reproductive Health 19, no. 1 (2022): 137, 10.1186/s12978-022-01449-z. 35698149 PMC9195231

[104] P. Liu , Y. Lu , R. Li , and X. Chen , “Use of Probiotic Lactobacilli in the Treatment of Vaginal Infections: In Vitro and In Vivo Investigations,” Frontiers in Cellular and Infection Microbiology 13 (2023): 1153894, 10.3389/fcimb.2023.1153894. 37077531 PMC10106725

[105] Z. Mei and D. Li , “The Role of Probiotics in Vaginal Health,” Frontiers in Cellular and Infection Microbiology 12 (2022): 963868, 10.3389/fcimb.2022.963868. 35967876 PMC9366906

[106] B. Foxman , J. V. Marsh , B. Gillespie , and J. D. Sobel , “Frequency and Response to Vaginal Symptoms Among White and African American Women: Results of a Random Digit Dialing Survey,” Journal of Womens Health 7, no. 9 (1998): 1167–1174, 10.1089/jwh.1998.7.1167. 9861594

[107] J. D. Jenks , C. I. Aneke , M. M. Al‐Obaidi , et al., “Race and Ethnicity: Risk Factors for Fungal Infections?” PLoS Pathogens 19, no. 1 (2023): e1011025, 10.1371/journal.ppat.1011025. 36602962 PMC9815636

[108] J. F. Peipert , K. L. Lapane , J. E. Allsworth , C. A. Redding , J. D. Blume , and M. D. Stein , “Bacterial Vaginosis, Race, and Sexually Transmitted Infections: Does Race Modify the Association?” Sexually Transmitted Diseases 35, no. 4 (2008): 363–367, 10.1097/OLQ.0b013e31815e4179. 18360319

[109] I. Naumova and C. Castelo‐Branco , “Current Treatment Options for Postmenopausal Vaginal Atrophy,” International Journal of Women's Health 10 (2018): 387–395, 10.2147/IJWH.S158913. PMC6074805 30104904

[110] C. S. Stika , “Atrophic Vaginitis: Atrophic Vaginitis and Local Therapies,” Dermatologic Therapy 23 (2010): 514–522, 10.1111/j.1529-8019.2010.01354.x. 20868405

[111] M. B. Mac Bride , D. J. Rhodes , and L. T. Shuster , “Vulvovaginal Atrophy,” Mayo Clinic Proceedings 85 (2010): 87–94, 10.4065/mcp.2009.0413. 20042564 PMC2800285

[112] C. Castelo‐Branco , M. J. Cancelo , J. Villero , F. Nohales , and M. D. Juliá , “Management of Post‐Menopausal Vaginal Atrophy and Atrophic Vaginitis,” Maturitas 52, no. S1 (2005): 46–52, 10.1016/j.maturitas.2005.06.014. 16139449

[113] M. I. Dothard , S. M. Allard , and J. A. Gilbert , “The Effects of Hormone Replacement Therapy on the Microbiomes of Postmenopausal Women,” Climacteric 26, no. 3 (2023): 182–192, 10.1080/13697137.2023.2173568. 37051868

[114] P. Cox and N. Panay , “Non‐Hormonal Treatments for Managing Vulvovaginal Atrophy/Genitourinary Syndrome of Menopause,” Climacteric 26, no. 4 (2023): 367–372, 10.1080/13697137.2023.2210283. 37199295

[115] M. Hickey , R. Baber , J. Eden , et al., “Safety and Effectiveness of a Novel Home‐Use Therapeutic Ultrasound Device for the Treatment of Vaginal Dryness in Postmenopausal Women: A Pilot Study,” Menopause 30, no. 4 (2023): 383–392, 10.1097/GME.0000000000002157. 36749915

[116] D. M. Lubián‐López , C. A. Butrón‐Hinojo , S. Menjón‐Beltrán , et al., “Effects of Non‐Ablative Solid‐State Vaginal Laser (SSVL) for the Treatment of Vulvovaginal Atrophy in Breast Cancer Survivors After Adjuvant Aromatase Inhibitor Therapy: Preliminary Results,” Journal of Clinical Medicine 12, no. 17 (2023): 5669, 10.3390/jcm12175669. 37685736 PMC10488849

[117] M. C. Meriggiola , P. Villa , S. Maffei , et al., “Vulvovaginal Atrophy in Women With and Without a History of Breast Cancer: Baseline Data From the PatiEnt satisfactiON studY (PEONY) in Italy,” Maturitas 183 (2024): 107950, 10.1016/j.maturitas.2024.107950. 38462385

